# Benzoxaboroles are structurally unique binders of eukaryotic translation initiation factor 4E

**DOI:** 10.64898/2026.02.24.707563

**Published:** 2026-02-25

**Authors:** Joshua B. Combs, D. Matthew Peacock, Gregory B. Craven, Sungwon Jung, Ying Chen, Sang M. Le, Jack Taunton, Kevan M. Shokat

## Abstract

Benzoxaboroles offer unusual reactivity and protein recognition for the development of small molecule drugs. Despite this potential, they are uncommon in drug discovery or in large fragment screening libraries. We synthesized a small series of structurally related benzoxaboroles containing a diazirine/alkyne tag to enable in-cell photoaffinity labeling (PAL) experiments. A subset of this library was found to have high selectivity for eukaryotic translation initiation factor 4E (eIF4E). The benzoxaborole–eIF4E interaction was found to be stereoselective in nature and competitive with the 7-methylguanosine cap of mRNA. Site of labeling experiments revealed that the benzoxaborole fragment interacts with the cap binding pocket of eIF4E. *In silico* modeling of the modified protein suggests that H-bonding interactions between the main chain of Trp102 and the side chain of Asn155 to the amide carbonyl and anionic boronate of the benzoxaborole, respectively, drive affinity for this challenging to drug pocket.

## Full Text Availability

The license terms selected by the author(s) for this preprint version do not permit archiving in PMC. The full text is available from the preprint server.

